# Integrated Analyses of Microbiome and Longitudinal Metabolome Data Reveal Microbial-Host Interactions on Sulfur Metabolism in Parkinson’s Disease

**DOI:** 10.1016/j.celrep.2019.10.035

**Published:** 2019-11-12

**Authors:** Johannes Hertel, Amy C. Harms, Almut Heinken, Federico Baldini, Cyrille C. Thinnes, Enrico Glaab, Daniel A. Vasco, Maik Pietzner, Isobel D. Stewart, Nicholas J. Wareham, Claudia Langenberg, Claudia Trenkwalder, Rejko Krüger, Thomas Hankemeier, Ronan M.T. Fleming, Brit Mollenhauer, Ines Thiele

**Affiliations:** 1 School of Medicine, National University of Galway, Galway, Ireland; 2 Luxembourg Centre for Systems Biomedicine, University of Luxembourg, Campus Belval, Esch-sur-Alzette, Luxembourg; 3 Department of Psychiatry and Psychotherapy, University Medicine Greifswald, Greifswald, Germany; 4 Division of Systems Biomedicine and Pharmacology, Leiden Academic Centre for Drug Research, Faculty of Science, Leiden University, Leiden, the Netherlands; 5 MRC Epidemiology Unit, University of Cambridge, Cambridge CB2 0QQ, UK; 6 Paracelsus-Elena-Klinik, 34128 Kassel, Germany; 7 Department of Neurosurgery, University Medical Center Goettingen, 37075 Goettingen, Germany; 8 Department of Neurology, University Medical Center Goettingen, 37075 Goettingen, Germany; 9 Centre Hospitalier de Luxembourg (CHL), Luxembourg, Luxembourg; 10 Division of Microbiology, National University of Galway, Galway, Ireland; 11 APC Microbiome Ireland, Ireland

**Keywords:** metabolism, Parkinson's disease, neurodegenerative disease, microbiome, transsulfuration pathway, metabolomics, metagenomics, metabolic modeling, taurine metabolism, bile acid metabolism

## Abstract

Parkinson’s disease (PD) exhibits systemic effects on the human metabolism, with emerging roles for the gut microbiome. Here, we integrate longitudinal metabolome data from 30 drug-naive, *de novo* PD patients and 30 matched controls with constraint-based modeling of gut microbial communities derived from an independent, drug-naive PD cohort, and prospective data from the general population. Our key results are (1) longitudinal trajectory of metabolites associated with the interconversion of methionine and cysteine via cystathionine differed between PD patients and controls; (2) dopaminergic medication showed strong lipidomic signatures; (3) taurine-conjugated bile acids correlated with the severity of motor symptoms, while low levels of sulfated taurolithocholate were associated with PD incidence in the general population; and (4) computational modeling predicted changes in sulfur metabolism, driven by *A. muciniphila* and *B. wadsworthia*, which is consistent with the changed metabolome. The multi-omics integration reveals PD-specific patterns in microbial-host sulfur co-metabolism that may contribute to PD severity.

## Highlights

• Longitudinal metabolomics reveal disturbed transsulfuration in Parkinson’s disease
• Metabolic modeling of gut microbiomes show altered microbial sulfur metabolism
• Changed microbial sulfur metabolism is linked to *B. wadsworthia* and *A. muciniphila*
• Taurine-conjugated bile acids are associated with incident Parkinson’s disease

## Graphical Abstract

**Figure:**
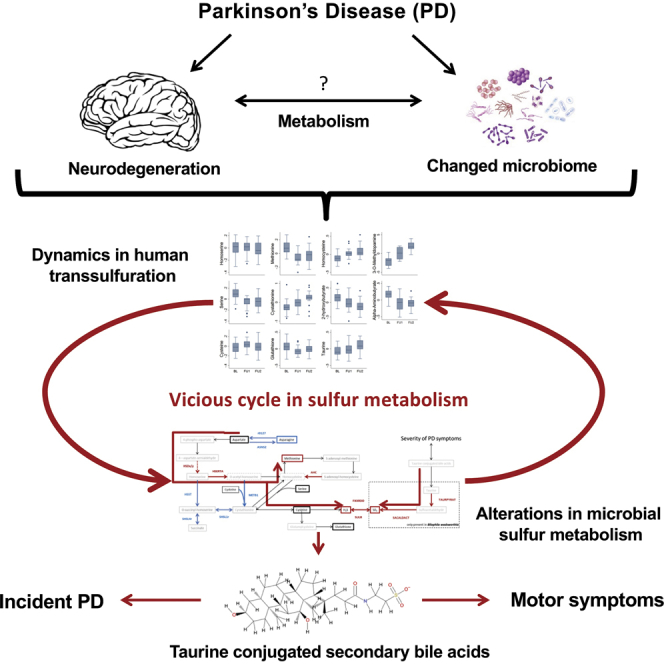

## Teaser

Hertel et al. demonstrate complex alterations in human and microbial sulfur metabolism in Parkinson’s disease by integrating longitudinal metabolomics and computational modeling of gut microbiomes. Then, potential clinical importance is revealed as secondary taurine-conjugated bile acids are shown to be associated with disease severity and Parkinson’s disease incidence.

## Introduction

Parkinson’s disease (PD) is a complex neurodegenerative disease with diverse underlying etiological paths and systemic consequences for patients’ physiology and metabolism (Kalia and Lang, 2015). Cumulative evidence suggests a contribution of peripheral metabolic factors, such as gut microbiome changes (Bedarf et al., 2017, Heintz-Buschart et al., 2018, Scheperjans et al., 2015), metabolic alterations (Havelund et al., 2017), and peripheral inflammation (Qin et al., 2016) to disease risk and progression (Mule and Singh, 2018, Sampson et al., 2016). Their causal role in the progression of the disease remains largely unknown, partly due to a lack of longitudinal human omics data. Such data could facilitate the investigation of the underlying disease dynamics, while controlling for important confounding factors, such as changes in drug regimens.

The contribution of PD-related microbiome changes to human metabolism in PD remains unknown. To integrate human metabolomic data with microbial abundance data, computational modeling approaches are required. The Constraint-Based Reconstruction and Analysis (COBRA) method is a pertinent computational modeling approach, already successfully used in various biomedical challenges (Aurich and Thiele, 2016). Condition-specific metabolic models can be derived through the application of condition-specific constraints, such as omics data (Yizhak et al., 2010) Capitalizing on metabolic reconstructions of hundreds of gut microbes (Magnúsdóttir et al., 2017), metagenomics data have been used to predict metabolic outputs of microbial community (Baldini et al., 2019, Heinken et al., 2017), which can be integrated with metabolomic data.

In this study, we first set out to determine PD-associated metabolic changes and disease progression by analyzing the plasma metabolome of 30 PD patients and 30 algorithmically matched controls at 3 time points. Second, we aimed at identifying the potential microbial contribution to the observed metabolic changes in PD at baseline. Therefore, we re-analyzed published metagenomic data from an independent PD cohort (Bedarf et al., 2017) consisting of 31 drug-naive PD patients and 28 age-matched controls using constraint-based metabolic modeling. With our analyses, we revealed PD-specific interactions of host-microbial metabolic activity on sulfur metabolism with relevance for the severity of motor symptoms.

## Results and Discussion

We obtained EDTA-plasma samples taken from the well-defined longitudinal DeNoPa cohort of initially drug-naive PD patients (n = 30) and matched healthy controls (n = 30; Table S1), each followed for 4 years, with samples taken every 2 years (Mollenhauer et al., 2013, Mollenhauer et al., 2016). Both groups remained comparable in basic physiological traits over time, such as BMI, basic blood indicators of kidney function, serum γ-glutamyl-transferase levels, and lipid status, while PD-related traits changed over time in the PD group (Tables S1 and S2).

**Figure 1:**
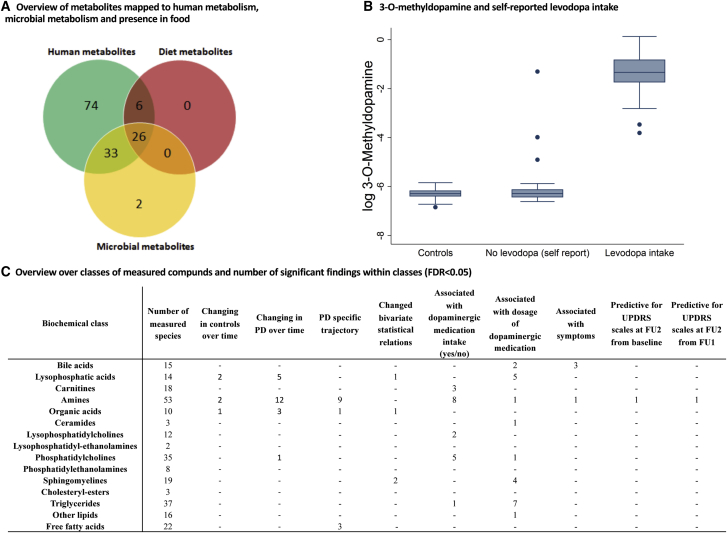
Overview over Metabolomic Analyses and 3-*O*-Methyldopamine Levels in Dependency on Levodopa Intake (A) Venn diagram represents the measured metabolites mapped onto the Virtual Metabolic Human database (www.vhm.life; Noronha et al., 2018) hosting the human metabolic reconstruction (Brunk et al., 2018), 818 microbial metabolic reconstructions (Magnúsdóttir et al., 2017), and the composition of >8,000 foodstuffs. (B) Levodopa intake (self-report) and 3-*O*-methyldopamine levels. (C) Overview of significant findings for biochemical classes for each line of analyses are shown.

Using a targeted metabolomic analysis, we measured 271 metabolites (Figure 1C), which were selected based on biomarkers and pathways implicated in neurodegeneration and PD (e.g., Fujita et al., 2014). Of those, 141 (52%) could be mapped onto human (Brunk et al., 2018) and gut microbial metabolic reconstructions (Magnúsdóttir et al., 2017) hosted in the Virtual Metabolic Human (VMH) database (Noronha et al., 2018) (Figure 1A). The remaining 130 unmapped metabolites were mostly lipids.

### The Levodopa-Degradation Product, 3-*O*-Methyldopa, Allows for Levodopa Intake Classification

We first investigated 3-*O*-methyldopa (3-OMD), the main catabolite of levodopa (Müller et al., 2002). According to self-reports, 16/30 cases took levodopa at follow up I and 24/29 cases at follow-up II (Table S1). The 3-OMD levels enabled the perfect classification of self-reported levodopa intake in follow-up assessments (area under the curve [AUC] = 1.00), making 3-OMD a useful marker for levodopa intake.

### The Longitudinal Metabolomic Data Reveal PD-Specific Trajectories

To identify time-dependent metabolites, we first analyzed the metabolome data separately for cases and controls. Only 5 metabolites changed over time in controls (Data S1). In contrast, 21 metabolites showed time dependency in PD after correction for multiple testing using the false discovery rate (FDR) (Benjamini, 2010), suggesting PD-specific dynamics in the plasma metabolome (Figure 1C) (Data S1). Testing trajectories of controls and PD cases directly against one another, we identified 3 types of PD-specific trajectories for 13 metabolites, which remained significant after correcting for multiple testing (Figure S1; see Method Details). Nine of these were also significantly different at baseline (Figure S1). The type 1 trajectory, only shown by homoserine, displayed constant levels in controls and increased concentrations in all 3 waves in PD. The type 2 trajectory (displayed by 3-OMD and cystathionine) had comparable metabolite levels at baseline between cases and controls, but increased over time in PD. Finally, the type 3 trajectory was defined by higher levels at baseline, compared to the controls, and decreased concentrations in PD in the follow-ups with no trend observed in the controls. The type 3 trajectory was found for 5 amino acids (methionine, serine, phenylalanine, leucine, and asparagine), 3 long-chain fatty acids (FA) (FA C14:0, FA C17:1, and FA C20:1), and the organic acids 3-hydroxyisobutyrate and α-aminobutyrate. Using principal-component analysis (PCA) of the type 3 metabolites, we found that the first PC displayed high loadings for all of the amines, while the second PC was primarily composed of the 3 long-chain FAs (Figure S1B). We conclude that the changes in amine metabolism were independent from those in lipid metabolism, despite having the same pattern over time (Figure S1A). All of the associations remained significant, except for 3-OMD and asparagine, when adjusting for medication (Data S1; Table S2).

### The Transsulfuration Pathway Is Altered in PD at Baseline and in Disease Progression

**Figure 2:**
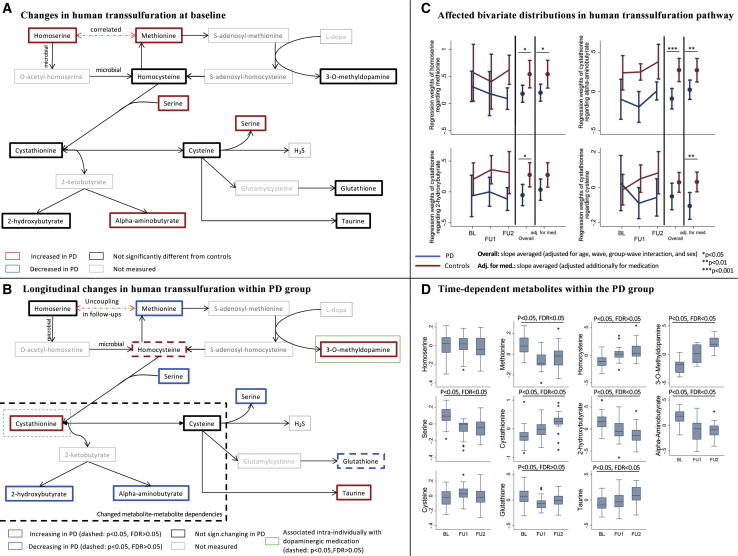
Longitudinal Alterations in the Human Transsulfuration Pathway in Association with PD (A) Changes in human transsulfuration pathway at baseline. Altered metabolite concentration (p < 0.05, FDR < 0.05 for difference in trajectory) in comparison to the control group are highlighted. (B) Longitudinal changes in transsulfuration within the PD group. Metabolites with significant trajectory within the PD group are highlighted (solid: p < 0.05, FDR < 0.05; dashed: p < 0.05, FDR > 0.05). (C) Altered metabolite-metabolite associations over the 3 waves in the transsulfuration pathway are shown. (D) Boxplots over the waves for all of the measured compounds in the transsulfuration pathway within the PD group are shown. The log concentrations were centered to the individual mean. BL, baseline; FU1, follow-up I; FU2, follow-up II.

All of the measured compounds within the transsulfuration pathway, except for cysteine, changed in concentration over time in PD with at least nominal significance (Figures 2A, 2B, and 2D). With homoserine, methionine, serine, and α-aminobutyrate being increased in PD compared to controls (Figure S1C), the PD-related effects were already prevalent at baseline. The transsulfuration compounds, however, belonged to different trajectory types. One possible explanation for these results is that metabolite-metabolite relations changed during PD progression. Thus, we investigated the statistical relations between homoserine (type 1 trajectory) and its downstream metabolite methionine (type 3 trajectory) in PD and controls over time. While methionine and homoserine were positively correlated in cases and controls, the controls had significantly higher regression coefficients than the PD patients (interaction effect [IE]: b = −0.36, 95% confidence interval [CI]: −0.66 to −0.06, p = 0.014; Figure 2C). Homoserine is not produced by human metabolism and is, for example, an intermediate of the microbial pathway generating methionine from aspartate. Hence, the gut microbiome may also contribute to human methionine plasma levels. Accordingly, it has been shown that probiotics could mitigate the effects of methionine depletion in mice on a choline- and methionine-deficient diet (Ye et al., 2018). Notably, both metabolites were statistically uncoupled in PD in follow-up II, but not in the controls (Figure 2C). Given the pivotal role of oxidative stress in PD (Singh et al., 2019), a higher flux toward transsulfuration to generate the antioxidant glutathione may explain both the decreasing methionine levels and the smaller variance contribution of homoserine to the methionine levels in follow-up.

In line with the hypothesis of a higher flux toward transsulfuration, cystathionine accumulated over time in PD patients. However, cysteine levels were unchanged over time, indicating that the conversion of cystathionine to cysteine, via the enzyme cystathionine-γ-lyase, may be blocked by a capacity limitation (e.g., saturated kinetics). Both metabolites were negatively correlated in PD in the follow-up assessments (Figure 2C), while a slightly positive correlation was observed in controls (Figure 2C). The corresponding IE was significant with and without adjusting for medication, displaying a stronger effect size with adjustment for levodopa dosage (b = −0.14, 95% CI: −0.25 to −0.04, p = 0.005). Accordingly, we observed changes in the dependencies between cystathionine and the downstream products of cystathionine degradation, 2-hydroxybutyrate (IE b = −0.33, 95% CI: −0.59 to −0.07, p = 0.013) and α-aminobutyrate (IE b = −0.37, 95% CI: −0.56 to −0.17, p = 2.98e−4) (Figure 2C). These observations indicate a lower variance contribution of cystathionine levels to its downstream products in PD, which is consistent with a capacity limitation in the conversion of cystathionine to cysteine. Consequently, cystathionine would accumulate, while the downstream concentrations of products of this degradation reaction would be either unchanged (e.g., cysteine) or decreased (e.g., α-aminobutyrate and 2-hydroxybutyrate) over time. Oxidative stress could inactivate the cystathionine-γ-lyase (Diwakar and Ravindranath, 2007). Alternatively, cystathionine-γ-lyase expression (Zhao et al., 2014) is modulated by *NRF2* expression, a key driver of the antioxidative response (Katsuoka et al., 2005). *NRF2* is induced by the transcription factor MAFF, whose expression is downregulated in PD (Oerton and Bender, 2017).

The ability to generate hydrogen sulfide from cysteine is an important feature of human cystathionine-γ-lyase activity (Bełtowski and Jamroz-Wiśniewska, 2014). While being potentially pro-inflammatory in the gastrointestinal tract, hydrogen sulfide has been shown to have neuroprotective attributes in PD pathology (Hu et al., 2010). As we found indications for reduced cystathionine-γ-lyase activity in PD (Figures 2B and 2C), the production of hydrogen sulfide may be disturbed, which in return may promote disease progression.

In conclusion, the transsulfuration pathway, central to the antioxidant response, showed complex alterations at baseline and over time. This result agrees well with the current understanding that PD is, at least in part, a disease driven by mitochondrial dysfunction (Singh et al., 2019).

### PD Affects Metabolite-Metabolite Dependencies

Next, we screened any pair of metabolites on PD-specific dependencies. We identified 2 significant metabolite pairs after correcting for multiple testing. For both pairs, the effects were stable over the 3 waves (Figures S2A and S2B). The 2 sphingomyelin species SM(d18:1/25:0) and SM(d18:1/25:1) were highly correlated in controls, but poorly correlated in PD cases (Figures S2A and S2B) (IE b = −0.53, 95% CI: −0.73 to −0.33, p = 3.23e−7, FDR < 0.05). As sphingomyelins are major components of cell membranes (Slotte, 2013), this result hints at different sphingomyelin distributions in cell membranes in PD and controls. The missing correlation in PD may be interpreted as the uncoupling of the 2 sphingomyelin species abundances in cell membranes. Numerous studies have implicated sphingolipid metabolism and genetic variants in associated genes (e.g., the sphingomyelin phosphodiesterase 1, *SMPD1*, Entrez Gene: 6609) in neurodegenerative diseases, including PD (Dodge, 2017, Foo et al., 2013). However, the role of individual sphingomyelin species in their interactions with other lipid species, such as cholesterols, and their distribution in cell membranes remains largely unknown (Róg and Vattulainen, 2014). As we report only plasma concentrations, inferences about the sphingomyelin distributions in the CNSs cannot be made.

Homocitrulline and lysophosphatidylcholine 16:0 showed positive correlations in controls and negative correlations in PD (Figures S2A and S2B) (IE: b = −0.34, 95% CI: −0.47 to −0.21, p = 3.14e−7, FDR < 0.05). As homocitrulline is a known marker for chronic, low-grade inflammation (Pietzner et al., 2017), a positive correlation with lysophosphatidylcholines may be expected, as lysophosphatidylcholines are known drivers of peripheral inflammatory states (Schmitz and Ruebsaamen, 2010). The reversal of the correlation may indicate that in PD, which has a peripheral inflammatory component (Qin et al., 2016), the systemic low-grade inflammation may not have the same effects as in non-PD individuals.

### Dopaminergic Medication Modulates the Transsulfuration Pathway

**Figure 3:**
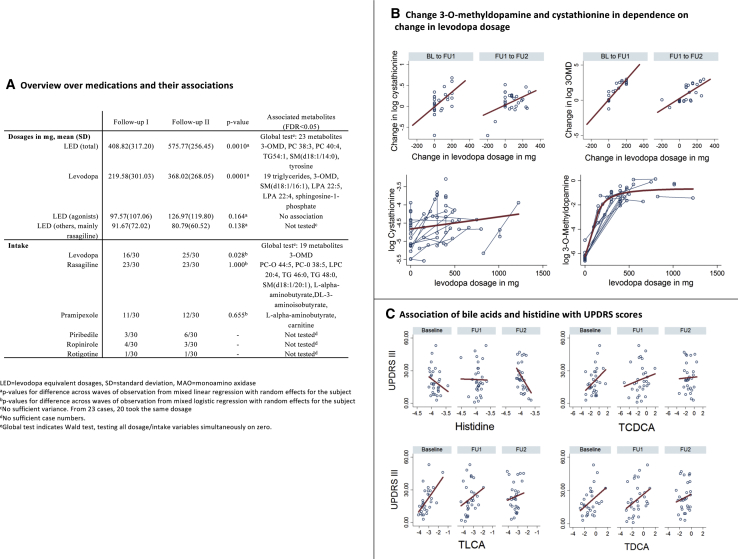
Signatures of Dopaminergic Medication in the Metabolome (A) Overview of results regarding dopaminergic medication, summarizing results significant after correcting for multiple testing (FDR < 0.05). Note that the global tests have no effect sign. (B) Change of 3-*O*-methyldopamine and cystathionine in dependency on change in levodopa dosage. In the lower panel, connected dots belong to the same individual. The lower panel shows that the change in cystathionine was not dependent on levodopa dosage, while for 3-*O*-methyldopamine, high levels led to less change. (C) Association of bile acids and histidine with UPDRS III scores (all FDR < 0.05). BL, baseline; FU1, follow-up I; FU2, follow-up II; PC, phosphatidylcholine; TG, triglyceride; UPDRS, Unified Parkinson’s Disease Rating Scale.

We investigated the effects of dopaminergic treatment as a potential causal factor for the observed dynamics in the transsulfuration pathway. We associated inter-individual metabolite level changes with corresponding changes in the administered levodopa equivalent dosages of dopaminergic medication (Figure 3A; Method Details). Among metabolites with PD-specific trajectories, only 3-OMD (b = 0.614, 95% CI: 0.377–0.851, p = 3.92e−7, FDR < 0.05) and cystathionine reached at least nominal significance (b = 0.078, 95% CI: 0.007–0.149, p = 0.030, FDR > 0.05). Investigating the different prevalent dopaminergic drugs, levodopa was the driving factor behind these associations (Figure 3B). However, we observed a saturation of 3-OMD levels (Figure 3B), indicating a capacity limitation to generate 3-OMD by catecholamine *O*-methyltransferase (COMT) activity, which also plays a role in, for example, epinephrine and norepinephrine degradation (Müller et al., 2002). Alternatively, a depletion of the *S*-adenosylmethionine, the major methyl donor for cellular methylation reactions, would be consistent with the observations.

In conclusion, the proposed higher flux through the transsulfuration pathway may be caused, at least in part, by levodopa medication. The question of whether a potential depletion of *S*-adenosylmethionine or COMT saturation may contribute to the PD-related pathology requires further investigation. Dopaminergic medication alone could not explain statistically the PD-specific trajectories of compounds belonging to the transsulfuration pathway, which were already prevalent at baseline.

### Dopaminergic Medication Has a Strong Lipodomic Signature

In the next step, we screened the metabolome for further associations with dopaminergic medication, using 2 analysis paradigms (Data S1; see Method Details). In intra-individual analyses, 2 lipids (phosphatidyl choline O-34:1 and triglyceride C58:9) passed the correction for multiple testing (all FDR < 0.05). In both cases, an intra-individual increase in equivalent dosage was associated with a decrease in metabolite concentration, hinting at drug-specific effects regarding the lipidomic changes (Data S1).

In combined inter-individual and intra-individual analyses, we identified 41 significant metabolite associations (FDR < 0.05; Figures 1C and 3A; Data S1). The metabolites included a range of different lipids, mainly phospholipid species and triglycerides, and amine breakdown products of phospholipids (e.g., ethanolamine, serine), constituting an impressive drug-specific signature in the lipidome. Levodopa dosage had the highest number of associations, 24, with 19 belonging to triglycerides (Figures 3A and S2D).

We observed associations between α-aminobutyrate and dopaminergic medication (MAO-B inhibitors and dopamine receptor agonists; Figure S2C). Comparable patterns could be observed in tendency (p < 0.05, FDR > 0.05) for other transsulfuration metabolites (methionine, cystathionine, and 2-hydroxybutyrate; Data S1). The effect sign of medication was inverse to the effect sign of longitudinal changes in these metabolites. However, due to the observational nature of our study, we could not differentiate between causes and consequences of drug treatments.

The profound lipidomic signature raises the question of whether dopaminergic treatment itself may contribute to the peripheral inflammation observed in PD. Dopamine can stimulate the release of adipokines, regulators of lipid metabolism and nutritional behavior (Blüher and Mantzoros, 2015), and pro-inflammatory cytokines by adipocytes (Wang, 2012). Adipokines are central regulators of lipid metabolism and, in the case of leptin, also nutritional behavior (Blüher and Mantzoros, 2015). Later stages of PD progression and usage of levodopa are often accompanied by weight loss (Akbar et al., 2015), which, based on our results, may be reflected in the lipidomic signature of dopaminergic medication. Consistently, levodopa treatment has been suggested as a predictor of weight loss (Wills et al., 2017). However, the dietary habits may change in response to dopaminergic medication, as noted by Aiello et al., (2015), contributing to lipidomic changes. Clarifying the nature of the associations between the lipidomic alterations and dopaminergic medication is therefore an intriguing target for future mechanistic research with potential clinical implications.

Our results suggest a potentially clinically relevant interplay of dopaminergic medication and PD with respect to the transsulfuration pathway. Finally, dopaminergic medication showed a strong and broad signature in the lipidome.

### Sulfur Metabolism Is Changed in PD-Associated Gut Microbial Communities

**Figure 4:**
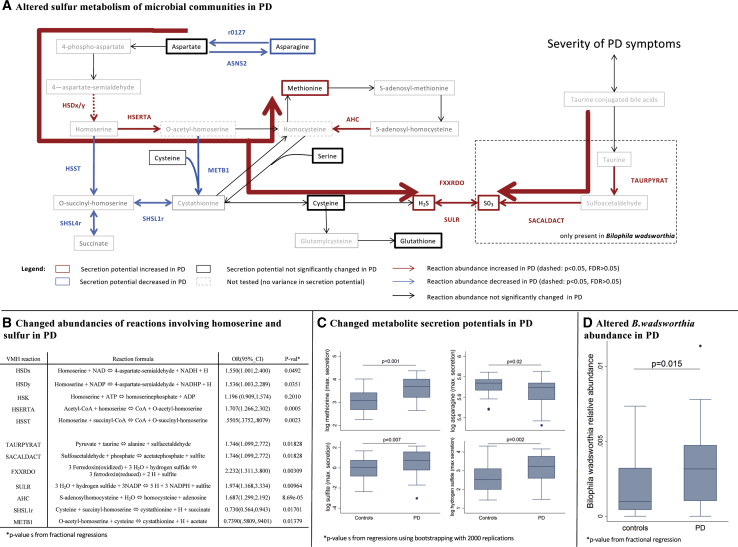
Results from Analyses of Individualized Microbiome Models (A) Depiction of microbial sulfur metabolism and generation of methionine from aspartate. The bold red arrow indicates the suggested increased overall flux toward final products. (B) Results of the statistical analyses of reaction abundances from fractional regressions. (C) Boxplots in PDs and controls for altered secretion potentials in the pathway depicted above. (D) Boxplots in PDs and controls for the abundance of *Bilophila wadsworthia*. Reaction abbreviations correspond to those in www.vmh.life.

To investigate the potential role of the gut microbes, we reanalyzed published metagenomic shotgun sequencing samples from 31 early-stage, levodopa-naive PD patients and 28 age-matched controls (Bedarf et al., 2017) using COBRA modeling. We created 59 personalized computational microbiome models based on these data (see Method Details). Of 5 microbial reactions involving homoserine, 4 were significantly changed in their abundances in the PD microbiome models (Figures 4A and 4B), which is consistent with the elevated homoserine levels in PD. Embedding these reactions in their biochemical context (Figure 4A) of generating methionine from aspartate, we computationally predicted the secretion flux potential for each of the pathways’ metabolites that could be transported by the microbes (see Method Details). The secretion potential of methionine, hydrogen sulfide, and sulfite was increased in PD microbiome models, while the asparagine secretion potential was decreased (Figures 4C and S3A; all FDR < 0.05). Accordingly, the abundances of 2 microbial sulfite reductases (EC 1.8.2.2 and 1.8.7.1) in the PD microbiome models were increased (Data S1).

In an analysis of the differential secretion potentials of 170 compounds, methionine was the top-hit passing correction for multiple testing (see Data S1). In addition, this screening revealed a strong overrepresentation of sulfur species in the significant results. While 5/10 sulfur-containing compounds had a p < 0.01, only 6/161 non-sulfur metabolites were significant, with a p < 0.01 (Fisher’s exact test: p = 0.004). The secretion potentials of cysteine-glycine, thiosulfate, and γ-aminobutyric acid (GABA) were also increased (FDR < 0.05). This latter observation should be investigated in future work, as it suggests that the microbiome could contribute differently to mammalian neurotransmitter production in PD.

Overall, our results demonstrate that the changes in microbial composition of PD gut microbiomes could cause altered microbial metabolic activity, which may be linked to the metabolism of the measured host.

### Increased Abundance of *Akkermansia muciniphila* and *Bilophila wadsworthia* Explains Microbial Sulfur Metabolism Capabilities

*A. muciniphila* and *B. wadsworthia* have been previously implicated in PD pathology as part of the microbial communities causal to the worsening of motor symptoms in genetically PD-predisposed mice (Sampson et al., 2016), although the underlying mechanisms have not yet been elucidated. Here, we linked the species abundance data to the computed secretion profiles. The *A. muciniphila* abundance contributed 71.5% (p = 2.197e−17) of the variance to the total methionine secretion potential, 59.0% (p = 7.735e−13) to the hydrogen sulfide secretion potential, and 49.4% (p = 3.381e−10) to the asparagine secretion potential. In contrast, the sulfite secretion potential was only slightly influenced by *A. muciniphila* (variance explanation: 16%, p = 0.001) (Figure S4B). We propose that *A. muciniphila* contributes to PD pathology via hydrogen sulfide production. Hydrogen sulfide, a highly reactive signaling molecule with multiple roles in humans (Wang, 2012), is pro-inflammatory (Singh and Lin, 2015) and harmful to the integrity of the mucus layer (Desai et al., 2016). Thus, the increased intestinal levels of hydrogen sulfide may contribute to the gastrointestinal problems associated with PD, such as constipation, and may lead to a higher absorption of bacterial toxins through a thinner gut barrier.

*B. wadsworthia* also showed an increased abundance in the PD microbiomes (control: mean abundance = 0.20%, PDs: mean abundance = 0.34%, p = 0.020; Figure 4D), while it contributed 22.0% (p = 0.0001) of variance to the total sulfite secretion potential. We propose that *B. wadsworthia* is a keystone species for sulfite production in the human gut microbiome. Accordingly, *B. wadsworthia* has been associated with gut inflammation in various mice models with implications for the host sulfur metabolism (Devkota et al., 2012, Natividad et al., 2018). Moreover, sulfite is a known neurotoxin affecting brain mitochondrial energy homeostasis (Grings et al., 2014), and its increase reduces brain cell glutathione levels (Zhang et al., 2004). Accordingly, we observed reduced blood glutathione levels in the follow-ups of the PD patients (Figure 2B). An increase in sulfite has been implicated in PD (Marshall et al., 1999), but it has not been connected to the human gut microbiome.

### Taurine-Conjugated Bile Acids Are Stable Markers of Variability in the Severity of Motor Symptoms

Finally, we hypothesized that the alterations in sulfur metabolism may contribute to an individual’s disease severity. Three taurine-conjugated bile acids (taurolithocholic acid [TLCA], taurodeoxycholic acid [TDCA], and taurochenodeoxycholic acid [TCDCA]) were positively associated across the waves with the Unified Parkinson’s Disease Rating Scale subscale III (UPDRS III) score (clinical motor examination, in which a lower score corresponds to better motor skills) and the glycine conjugate of chenodeoxycholic acid and histidine (FDR < 0.05, Figure 3C; Table S3; Data S1). In agreement, bile acid metabolism has been implicated in neurodegenerative diseases, including PD (Ackerman and Gerhard, 2016).

In the liver, bile acids are conjugated with either taurine, a final breakdown product of the transsulfuration pathway (Figures 2 and 3), or glycine (Alnouti, 2009). Subsequently, they are transported into the gastrointestinal tract, where the gut microbiota can deconjugate them. Based on our results, we propose a human-microbial transsulfuration cycle driven by the continuous removal of taurine by *B. wadsworthia* and sulfur metabolite production by *A. muciniphila* and *B. wadsworthia.* We also hypothesize that the longitudinally stable association between taurine-conjugated bile acids and UPDRS scores is furthered by *B. wadsworthia* abundance-mediated peripheral inflammation. In this context, it is worth noting that taurine itself is an inhibitory neurotransmitter. Produced and secreted by neurons in various scenarios of stress, including mitochondrial dysfunctions, taurine appears to increase the chance of neuronal survival by regulating calcium influx and stimulating the expression of antioxidant genes (Saransaari and Oja, 2007, Wu et al., 2009). Accordingly, taurine has been reported to be increased in the brain in mice after the injection of α-synuclein (Graham et al., 2018). These experimental studies emphasize the importance of sulfur metabolism in the CNS in general and taurine in particular.

In conclusion, sulfur metabolism is profoundly changed in PD in interaction with gut microbiota, namely *A. muciniphila* and *B. wadsworthia*. We provided evidence that these changes may translate via taurine-conjugated bile acids into variability in severity of clinical symptoms based on the UPDRS subscale III in PD. Beyond PD, the interplay between microbiota, especially *B. wadsworthia*, and mammalian transsulfuration could have implications for other diseases related to oxidative stress response, as has already been shown for metabolic disorders in mice models (Devkota et al., 2012, Natividad et al., 2018).

### Sulfated Taurolithocholic Acid Associates with PD Incidence in the General Population

As the aforementioned analyses had not been designed to provide insight into a possible role for these compounds in disease etiology, we extended our analysis by investigating whether taurine-conjugated bile acids are associated with PD incidence. Therefore, we used a large, prospective general population cohort, the European Prospective Investigation of Cancer, Norfolk (EPIC-Norfolk) (Day et al., 1999). In EPIC-Norfolk, untargeted metabolome data were available from stored baseline samples of 10,034 individuals, 157 of whom have been subsequently diagnosed with PD based on either hospital admission records or death certificates during >20 years of follow-up. We used Cox proportional hazards regression analyses to test associations between 7 different taurine-conjugated bile acids and PD incidence, adjusting for age, sex, BMI, smoking, and plasma abundances of C-reactive protein (Data S1). Taurolithocholate 3-sulfate was significantly associated with a reduced risk of developing PD after accounting for multiple testing (hazard ratio per SD = 0.80, 95% CI: 0.69 to 0.94, p = 0.0055). The hepatic sulfation of secondary bile acids serves as a detoxification mechanism by increasing the water solubility of bile acids and, subsequently, their clearance via filtration (Alnouti, 2009). In addition, the reuptake of sulfated bile acids by the liver is less efficient, which limits in return their enterohepatic recirculation (Alnouti, 2009, Gärtner et al., 1990). Note that only taurolithocholic acid (desulfated) showed a significant relation to the symptoms in the PD cases (Figure 3C). These results indicate that PD-relevant host-microbial interactions in sulfur and, interlinked, bile-acid metabolism may play a role in the development of the disease.

### Limitations

In our study, the effects of exercise were not monitored. However, the metabolomic signature of exercise as reported in the literature (Daskalaki et al., 2015) does not overlap with our reported changes in transsulfuration, and in early PD, no major differences to controls in exercise could be recorded (Mantri et al., 2018). In addition, we cannot rule out that our results were influenced by dietary variance, although it seems unlikely given the high homogeneity in the case-control design. Moreover, the associations between the lipidomic alterations and dopaminergic medication require further investigations. Longitudinal studies integrating several layers of metabolome data (fecal, blood, and urine) with metagenomics data within the same individuals are needed to corroborate and extend our results. Finally, being explorative in nature, our metabolomic analyses are not suitable for mechanistic insights, but are only able to provide a holistic description of metabolic alterations facilitating novel hypothesis generation.

### Conclusion

The systemic nature of PD strongly manifests in the metabolomic trajectories over time. Analyzing these PD-specific trajectories in combination with the metagenomics data of gut microbial communities opens up new research routes toward a better understanding and prediction of phenotypic variability in PD.

## STAR★Methods

### Key Resources Table

**Table:** 

REAGENT or RESOURCE	SOURCE	IDENTIFIER
**Biological Samples**		

EDTA-plasma samples DeNoPa	Mollenhauer et al., 2013	https://www.denopa.de/

**Critical Commercial Assays**		

DiscoveryHD4® platform	Metabolon Inc.	N/A

**Deposited Data**		

Shotgun sequencing data stool samples	Bedarf et al., 2017	ERP019674
Genome scale reconstructions (AGORA v1.02)	Magnúsdóttir et al., 2017	vmh.life
Summary statistics	This Paper	Data S1

**Software and Algorithms**		

COBRA Toolbox	Heirendt et al., 2019	https://opencobra.github.io/
Microbiome Modeling Toolbox	Baldini et al., 2019	https://github.com/opencobra/cobratoolbox/tree/master/src/analysis/multiSpecies/microbiomeModelingToolbox/
Rsamtools(v1.32.0)	Morgan et al., 2019	https://rdrr.io/bioc/Rsamtools/
Burrows-Wheeler-Aligner software	Li and Durbin, 2009	http://bio-bwa.sourceforge.net/
Samtools	Li et al., 2009	http://samtools.sourceforge.net/
IBM CPLEX	IBM Inc.	N/A
R-Studio	N/A	https://www.r-project.org/
STATA 14/MP	STATA Inc.	N/A
MATLAB v2016b	Mathworks Inc.	N/A

**Other**		

Leiden Targeted Metabolomics Platform	Leiden University BioMedical Metabolomics Facility	https://www.universiteitleiden.nl/en/research/research-facilities/science/biomedical-metabolomics-facility-leiden
Data from the EPIC-Norfolk study	Day et al., 1999	http://www.srl.cam.ac.uk/epic/contact/index.shtml
Statistical and computational scripts	This paper	https://github.com/ThieleLab/CodeBase/tree/master/Scripts_Hertel_CellReports_2019
Clinical and metabolomic data from the DeNoPa study	This paper	brit.mollenhauer@paracelsus-kliniken.de

### Lead Contact and Materials Availability

Further information and requests for resources and reagents should be directed to and will be fulfilled by the Lead Contact, Ines Thiele (ines.thiele@nuigalway.ie). This study did not generate new unique agents.

### Experimental Model and Subject Details

#### Study participants from the De Novo Parkinson’s disease cohort (DeNoPa)

Samples and data of PD subjects and healthy controls were part of the longitudinal *de novo* Parkinson’s disease (DeNoPa) cohort (Mollenhauer et al., 2013, Mollenhauer et al., 2016). Recruitment was initiated between 2009 and 2010 at the Paracelsus-Elena-Klinik, Kassel, Germany, and consisted of 110 healthy controls and 159 *de novo* PD patients with frequency matching of cases and controls regarding age, sex, and education. Inclusion and exclusion criteria have been previously described in detail (Mollenhauer et al., 2013, Mollenhauer et al., 2016). All PD subjects had to fulfill *de novo* criteria with L-DOPA exposure no longer than 2 weeks and not within 4 weeks prior to study entry (as in the ADAGIO trial (Olanow et al., 2008)). In brief, participants were between 40 and 85 years old, without known vascular encephalopathy, hydrocephelaus, multiple system atrophy, and progressive supranuclear palsy. Healthy controls were, in addition, without known or treated psychiatric or neurological conditions. Subjects were followed biannually. Plasma samples were drawn in the morning fasting with BD Vacutainer and processed as published. The study was conducted according to the Declaration of Helsinki and with informed written consent provided by all subjects. The study was approved by the ethics committee of the Physician’s Board Hessen, Germany (Approval No. FF89/2008) and has been registered at the German Register for Clinical trials (DRKS00000540) according to the WHO Trial Registration Dataset.

#### Computationally optimized sample matching and selection in DeNoPa

For the metabolomic characterization, 30 PD patients and 30 controls were selected from the DeNoPa cohort matched for age, sex, body mass index, and multiple comorbidities. EDTA-plasma samples (500 mL each) from the baseline assessment, the follow-up I (24 months), and the follow-up II (48 months) were obtained and subjected to metabolomics characterization. For one control participant, metabolomic characterization failed at the baseline due to missing biomaterial resulting in 179 metabolomic profiles in total.

In order to minimize the influence of confounding factors on the metabolomics measurements and remove unwanted sources of variation, we applied multiple filtering steps to the biospecimen collection for the DeNoPa cohort and computationally optimized the matching and selection of the 30 patients and 30 control biospecimens used for metabolic profiling.

First, in order to reduce variation in the biospecimen-derived data due to the presence of non-representative genetic forms of PD, we filtered out all samples from subjects with known PD-associated genetic alterations in the genes GBA, PRKN and DJ1, as well as subjects with the SNCA REP1 263bp promotor variant (Ritz et al., 2012). For the remaining samples from the cohort, the selection was optimized by computationally searching for a selection of 30 patients and 30 controls that best meets the following criteria: (1) the gender representation should be balanced and matched across patients and controls; (2) the age and body mass index (BMI) distributions should be as similar as possible for selected patients and controls (measured using the Kolmogorov-Smirnov Test); (3) to reduce influences of medication and comorbidities on the metabolomics measurements, the number of subjects in the selection who used medication for blood pressure related issues or other medical symptoms should be minimized; and (4) the number subjects with known common genetic variations that may affect PD-risk or PD-related symptoms, in particular subjects with the SNCA polymorphism rs1193107 (Liu et al., 2018) or with one or two APOE E4 alleles, which cannot not be filtered out completely due to their frequent occurrence, should be minimized in the selection. In order to optimize the sample selection for these criteria, the prefiltered set of biospecimen available in for the cohort was filtered in a recursive feature elimination procedure by iteratively removing the samples, whose exclusion maximally improved the current selection. For this purpose, sample selections were scored within the iterative procedure by quantifying the deviations from the theoretically achievable optimum for each criterion and computing the sum of these deviations (except for the gender representation, which was required to be optimally matched and balanced, all other criteria entered the sum of deviations score with an equal weight). Information on age, sex, and further basic covariates can be found in Table S1.

#### Study participants from the EPIC-Norfolk study

The EPIC-Norfolk study is a cohort of 25,000 individuals aged between 40 and 79 at recruitment, from the general population of Norfolk (East England) (Day et al., 1999), nested within the European Prospective Investigation into Cancer and Nutrition (EPIC). The study was approved by the Norwich Local Ethics Committee (previously known as Norwich District Ethics Committee; REC Ref: 98CN01) and all participants gave their written consent before entering the study. Untargeted metabolomics were measured using the DiscoveryHD4® platform (Evans et al., 2014) (Metabolon, Inc., Durham, USA) in non-fasted citrated plasma samples, in two quasi-randomly selected batches. Metabolite levels were median-normalized across run days and no imputation of missing values was performed. Prior to statistical analyses, metabolite levels were natural log transformed, winsorised (to 5 standard deviations (SD)) and standardized (μ = 0, SD = 1). Processing was performed for each batch separately. The reported analyses included 10,034 individuals with full covariate information and metabolomic quantifications measured. The mean in age was 59.8(SD = 8.9) and 53% of the participants were female. Hospitalisation data were obtained using National Health Service numbers through linkage with the East Norfolk Health Authority (ENCORE) database, which contains information on all hospital contacts throughout England and Wales. Participants were identified as incident cases if the corresponding ICD-9 or ICD-10 code was registered as the cause of hospitalisation, or on the death certificate (as the underlying cause of death or as a contributing factor). The current study includes follow-ups until 31st March 2016. The dataset contained 157 incident PD cases with a mean time to event of 13.9 years (SD 4.7), while prevalent PD cases based on self-reported medication reports were excluded.

### Method Details

#### Procedures and measurements of phenotyping in DeNoPa

The complete protocol of all procedures is reported elsewhere (Mollenhauer et al., 2013), including comprehensive neuropsychiatric testing, clinical assessments, sampling of biomaterial, and biobanking. Using a standardized protocol, assessments were carried out in cases and controls in the same order in all three waves of observations. In this study, we focused on the core symptoms of PD measured by the revised Unified Parkinson’s Disease Rating Scale (Goetz et al., 2007), consisting of four subscales (I: non-motor experiences of daily living; II: motor experience of daily living; III: motor examination; and IV: motor complications). For the initial screening of metabolic biomarkers, we also utilized the total sum of all four scores as an overall indicator for the severity of PD symptoms.

#### Medication data in DeNoPa

By design of the DeNoPa study, all PD patients were drug-naive at baseline assessments, being treated subsequently with dopaminergic modulators according to German S3-guidelines. At baseline, the study protocol ensured that patients stopped levodopa intake at least four weeks prior enrolment (Mollenhauer et al., 2013, Mollenhauer et al., 2016). Nonetheless, we observed three cases at baseline with at least 10-fold increased 3-OMD levels compared to drug-naive PD patients (Figure 1B). However, two of these cases exhibited lower 3-OMD levels than most of the levodopa-treated patients (increase up to 600-fold), such that it is unclear whether these patients were taking levodopa at baseline against the study protocol or whether the time-period of four weeks was long enough to wash out all levodopa related metabolites. Plausible alternative explanations include variation in genes involved in dopamine metabolism (Jiménez-Jiménez et al., 2014, Klebe et al., 2013), or metabolic activity of the gut microbiome, which can produce dopamine and dopamine precursor (Belik et al., 2017) In the follow-up assessments, the daily doses of levodopa and the equivalent dosages (according to (Tomlinson et al., 2010)) were recorded at the day of blood sampling. These reports were compared with the 3-OMD levels from metabolomic analyses, revealing a perfect classification (AUC = 1) of levodopa intake by 3-OMD in the follow-up assessments. However, at baseline, three PD patients showed strongly increased 3-OMD levels, indicating that they took levodopa medication while being self-reported drug-naive. Further, one observation showed missing values in the dosage variable, while having strongly increased 3-OMD levels. These four observations were re-classified as ‘levodopa-treated’ for statistical analyses or, if information about dosage was required, excluded.

#### Metabolomic Measurements in DeNoPa

Metabolomic measurements were performed at the Leiden University BioMedical Metabolomics Facility, using previously described and validated mass-spectrometric (MS) based platforms.

##### Bile acid profiling

50 μL of each plasma sample was spiked with internal standard solutions. The extraction of the bile acids compounds is performed by protein precipitation with methanol. After collection, the supernatant is concentrated by first drying and then reconstituted in a smaller volume. After reconstitution, the extract is transferred into amber autosampler vials for analysis. A Shimadzu system formed by three high pressure pumps (LC-30AD), controller (CBM-20Alite), auto sampler (SIL-30AC) and an oven (CTO-30A) from Shimadzu Benelux, was coupled online with a LCMS-8050 triple quadrupole mass spectrometer (Shimadzu) operated using LabSolutions data acquisition software (Version 5.89, Shimadzu). The samples were analyzed by UPLC-MS/MS using an Acquity UPLC HSS T3 column (Waters). The triple quadrupole mass spectrometer was used in negative ion mode and all analytes were monitored in dynamic Multiple Reaction Monitoring (dMRM).

##### Acylcarnitine profiling

10 μL of each sample was spiked with an internal standard solution. Then proteins were precipitated by the addition of MeOH. The supernatant was transferred to an autosampler vial. The vials were transferred to an autosampler tray and cooled to 10°C until the injection. 1.0 μL of the sample mixture was injected into the triple quadrupole mass spectrometer. Chromatographic separation was achieved by UPLC (Agilent 1290, San Jose, CA, USA) on an AccqTag Ultra column (Waters) with a flow of 0.7 mL/min over a 11 min gradient. The UPLC was coupled to electrospray ionization on a triple quadrupole mass spectrometer (Agilent 6460, San Jose, CA, USA). Analytes were detected in the positive ion mode and monitored in Multiple Reaction Monitoring (MRM) using nominal mass resolution. The acquired data were evaluated using Agilent MassHunter Quantitative Analysis software (Agilent, Version B.05.01), by integration of assigned MRM peaks and normalization using proper internal standards. The closest-eluting internal standard was employed. In-house developed algorithms were applied using the pooled QC samples to compensate for shifts in the sensitivity of the mass spectrometer over the batches.

##### Amine profiling

The amine platform covers amino acids and biogenic amines employing an Accq-tag derivatization strategy adapted from the protocol supplied by Waters. 5 μL of each sample was spiked with an internal standard solution. Then proteins were precipitated by the addition of MeOH. The supernatant was transferred to a new Eppendorf tube and taken to dryness in a speedvac. The residue was reconstituted in borate buffer (pH 8.5) with AQC reagent. After reaction, the vials were transferred to an autosampler tray and cooled to 4°C until the injection. 1.0 μL of the reaction mixture was injected into the UPLC-MS/MS system. Chromatographic separation was achieved by an Agilent 1290 Infinity II on an Accq-Tag Ultra column (Waters) with a flow of 0.7 mL/min over a 11 min gradient. The UPLC was coupled to electrospray ionization on a triple quadrupole mass spectrometer (AB SCIEX Qtrap 6500). Analytes were detected in the positive ion mode and monitored in Multiple Reaction Monitoring (MRM) using nominal mass resolution. Acquired data were evaluated using MultiQuant Software for Quantitative Analysis (AB SCIEX, Version 3.0.2), by integration of assigned MRM peaks and normalization using proper internal standards. For analysis of amino acids their 13C15N-labeled analogs were used. For other amines, the closest-eluting internal standard was employed. Blank samples were used to determine blank effect. Inhouse developed algorithms were applied using the pooled QC samples to compensate for shifts in the sensitivity of the mass spectrometer over the batches.

##### Oxidative stress profiling

The oxidative stress platform is divided in two chromatographic methods: low and high pH. In the low pH method, isoprostanes, prostaglandins, nitro-fatty acids and lyso-sphingolipids are analyzed. The high pH method covers lyso-sphingolipids, lysophosphatidic acids, alkyl-lysophosphatidic acids and cyclicphosphatidic acids. 150 μL of each plasma sample was spiked with antioxidant and internal standard solutions. To extract the analytes from the aqueous phase, butanol and ethyl acetate are used. After collection, the organic phase is concentrated by first drying and then reconstituted in a smaller volume. After reconstitution, the extract is transferred into amber auto sampler vials and used for high and low pH injection, respectively. A Shimadzu system formed by three high pressure pumps (LC-30AD), a controller (CBM-20Alite), and autosampler (SIL-30AC) and an oven (CTO-30A) from Shimadzu Benelux, was coupled online with a LCMS-8050 triple quadrupole mass spectrometer (Shimadzu) operated using LabSolutions data acquisition software (Version 5.89, Shimadzu). The samples were analyzed by UPLC-MS/MS using a Kromasil Eternity XT C18 column (Akzo Nobel) for high pH and an Acquity BEH C18 column (Waters) for the low pH method. The triple quadrupole mass spectrometer was used in polarity switching mode and all analytes were monitored in dynamic Multiple Reaction Monitoring (dMRM). Sphingosines C17:1 and C18:1, Sphinganines C17:0 and C18:0, PAF C16:0 and PAF C16:0-d4 were measured in positive ion mode. The other metabolites were detected in negative mode. The acquired data was evaluated using LabSolutions software (Shimadzu), by integration of assigned MRM peaks and normalization using accordingly selected internal standards. When available, a deuterated version of the target compound was used as internal standard. For the other compounds, the closest-eluting internal standard was employed. An in-house written tool is applied using the QC samples to compensate for shifts in the sensitivity of the mass spectrometer throughout the batches. Both internal standard correction and QC correction were applied to the dataset.

##### Organic acid profiling

Sample preparation was done by doing first protein precipitation of 50 uL of plasma using MeOH/H2O with ISTD added. After centrifugation and transferring the supernatant, the samples evaporated to complete dryness on the speedvac. Then, two-step derivatization procedures were performed on-line: oximation using methoxyamine hydrochloride (MeOX, 15 mg/mL in pyridine) as first reaction and silylation using N-Methyl-N-(trimethylsilyl) trifluoroacetamide (MSTFA) as second reaction were carried out. 1 μL of each sample was injected directly after its derivatization on GC-MS. The metabolites were measured by gas chromatography on an Agilent Technologies 7890A equipped with an Agilent Technologies mass selective detector (MSD 5975C) and MultiPurpose Sampler (MPS, MXY016-02A, GERSTEL). Chromatographic separations were performed on a HP-5MS UI (5% Phenyl Methyl Silox), 30 m × 0.25 m ID column with a film thickness of 25 μm, using helium as the carrier gas at a flow rate of 1,7 mL/min. A single-quadrupole mass spectrometer with electron impact ionization (EI, 70 eV) was used. The mass spectrometer was operated in SCAN mode mass range 50- 500. The raw data were pre-processed using Agilent MassHunter Quantitative Analysis software (Agilent, Version B.05.01). In-house developed algorithms were applied using the pooled QC samples to compensate for shifts in the sensitivity of the mass spectrometer over the batches.

##### Positive lipid profiling

For positive lipid measurements in plasma samples, 1000 μL isopropyl alcohol containing internal standards were added to 10 μL plasma sample. Samples were centrifuged and supernatant was transferred to vials for LC-MS analysis. 2.5 μL was injected on a ACQUITY UPLC (Waters, Ettenleur, the Netherlands) with a HSS T3 column (1.8 μm, 2.1 ^∗^ 100 mm) with a flow of 0.4 mL/min over a 16 min gradient. The lipid analysis is performed on a UPLC-ESI-Q-TOF (Agilent 6530, Jose, CA, USA) high resolution mass spectrometer using reference mass correction. Lipids were detected in full scan in the positive ion mode. The raw data were pre-processed using Agilent MassHunter Quantitative Analysis software (Agilent, Version B.04.00). The lipid response was calculated as the peak area ratios of the target analyte to the respective internal standard.

##### Negative lipid profiling

For negative lipid analysis, 50 μL of plasma sample was used. 50 uL of internal standard solution was added after which precipitation of the proteins was carried out by adding 550 uL of MeOH. After precipitation of the proteins and centrifugation, 600 uL of supernatant was transferred and dried. The reconstitution step was done by adding 300 uL of isopropanol with 0.1% formic acid. The prepared samples were transferred to vials for LC-MS analysis. 8 μL was injected in total for analysis. The lipid analysis is performed on a ACQUITY UPLC (Waters, the Netherlands) coupled to a high resolution mass spectrometer with a Synapt G2 Q-TOF system (Waters, the Netherlands) using reference lock mass correction. Lipids were detected in full scan in the negative ion mode. Chromatographic separation was achieved using a HSS T3 column (1.8 μm, 2.1 ^∗^ 100 mm) with a flow of 0.4 mL/min over a 16 minute gradient. The raw data was pre-processed using Targetlynx software (Masslynx, V4.1, SCN916). The lipid response was calculated as the peak area ratios of the target analyte to the respective internal standard.

The QC-RSD and full descriptive statistics of the metabolome data, including data-base identifier, can be found in Data S1.

#### Mapping of metabolites from metabolomics data from the DeNoPa study onto the Virtual Metabolic Human database

Using the metabolite identities (names, HMDB IDs, lipid maps IDs) accompanying the metabolomic data, we translated, manually and automatically, the metabolites into the corresponding VMH IDs using the VMH’s API and the query interface (Noronha et al., 2018). Using the API, we identified all metabolites being part of the human metabolic reconstruction (Brunk et al., 2018), the gut microbial reconstructions (Magnúsdóttir et al., 2017), and the composition of the food stuff (US Department of Agriculture, Agricultural Research Service Program; Finley and Klurfeld, 2013).

#### Computing individual resolved strains relative abundance

Published metagenomic data from a cohort of early-stage, drug-naive, male Parkinson patients (n = 31) and age-matched, healthy controls (n = 28) (Bedarf et al., 2017) was obtained from the European Bioinformatics Institute-Sequence Read Archive database: ERP019674. For each individual, we combined the corresponding fastq files into one file. To obtain individual resolved strain abundances, we used the same protocol as described elsewhere (Bauer and Thiele, 2018). Briefly, the genomes of the 773 gut microbes, for which genome-scale metabolic reconstructions were available at the VMH database (www.vmh.life, v1.02), were obtained from KBase (https://kbase.us/) and NCBI Genome (https://www.ncbi.nlm.nih.gov/genome/) and combined into one file, such that each genome corresponds to a chromosome, representing the reference genome. Using Burrows-Wheeler Aligner (BWA) software (Li and Durbin, 2009) with default parameters, the paired reads were mapped to the human genome (version 38) to exclude human contaminant sequences. Subsequently, the metagenomic reads were mapped onto the reference genome using BWA with default parameters. Samtools (Li et al., 2009) was used to identify mapped reads discarding reads with low quality score and cross-mapped reads: coverage per genome (number of reads bases mapping the genome divided by the genome length) was computed with R package “Rsamtools” (Morgan et al., 2019). A minimal value of 0.1 of coverage (10%) was considered as a lower threshold for assessing microbial presence, reducing the number of false positives. Finally, relative abundances were retrieved for each individual microbiome, normalizing the total sum of microbial abundances to one.

#### Constructing and simulating individualized gut microbiota models

The genome-scale gut microbial reconstructions were downloaded from the VMH database (version AGORA 1.02 - Unconstrained). We then used the mgPipe module of the Microbiome Modeling Toolbox (Baldini et al., 2019) to create personalized microbiota models for each of the 59 individuals. Briefly, mgPipe combines all 773 microbial reconstructions into one microbial community reconstruction by placing them into one lumen compartment ([lu]), from which they can take up nutrients or secrete into by-products (Thiele et al., 2013). Dietary inputs (through reactions, such as ‘Diet_EX_glc_D[d]’ for D-glucose) are given into this lumen compartment and byproducts not been used by any community member are excreted from this lumen compartment using the defined exchange reactions (e.g., ‘EX_ac[fe]’ for acetate excretion). The community reconstruction also contains a community biomass reaction. The stoichiometric factors of this community biomass reaction were adjusted based on the determined relative abundances of each strain in a given metagenome. Strains that could not be detected in a sample, were removed from the corresponding individual microbial community model to reduce computation time. For all personalized microbial community models, a standard European diet (Noronha et al., 2018) was applied as constraints. The community biomass reaction for each personalized model was constrained to a lower bound > = 0.4 per day and an upper bound ≤ 1 per day, which corresponds to fecal excretion from at least every 3 days and at most once a day. For each personalized gut microbiota model, we then determined the next maximal secretion profiles as the absolute value of the difference between maximal secretion and uptake of all the compounds associated with uptake and excretion reactions in the model using flux balance analysis (Orth et al., 2010). All computations were performed in MATLAB version 2016b (Mathworks, Inc.), using the COBRA Toolbox (Heirendt et al., 2019) (commit: b097185b641fc783fa6fea4900bdd303643a6a7e) and the Microbiome Modeling Toolbox (Baldini et al., 2019). For solving the linear programming problems underlying the flux balance analysis, we used the IBM CPLEX (IBM, Inc.) solver through the Tomlab (Tomlab, Inc.) interface.

### Quantification and Statistical Analysis

#### Statistical analyses on DeNoPa data

For descriptive statistics, metric variables were described by means and standard deviations, while nominal variables were described by proportions. Additionally, intra-class correlations (ICC) were calculated, stratified for study group from mixed effect generalized linear models including the wave as predictor with random intercept for the subjects. The only variable with missing values was the levodopa dosage variable (n = 4). These observations were excluded from analyses, whenever the dosage was included into analyses. Note that in the case of mixed effect modeling only the observation was dropped but not the individual.

All p values are reported two-tailed and variables were controlled for outliers. Observations with more than four standard deviations away from the mean were excluded from analyses. Furthermore, significant findings were visually inspected via box-plots and distributional plots to lessen the chance of false positives by undetected outliers. Statistical analyses were performed in STATA 14/MP (College Station, Texas, USA). Summary Statistics of the performed analyses are given in the Supplemental data file Data S1. Note that although 271 metabolites were quantified 272 metabolite concentrations were analyzed. One metabolite (spingosine) was measured by two different methods.

#### Analyses of PD trajectories

To identify PD-specific trajectories in the metabolome, a series of mixed effect linear regression models were fitted with random intercepts for study participants and the log metabolite concentration being the response variable. The mixed effect models were fitted using generalized least-squares and heteroscedastic robust standard errors. First, we fitted models stratified for PD patients and controls testing on time-dependent metabolites within both study groups. These regressions were performed with the wave (categorical) as predictor of interest, adjusted for age and sex. In the next step, we tested for PD-specific trajectories in a combined analysis. We included group, wave (categorical), group-wave interaction terms as predictors of interest, adjusting for age and sex to reduce residual variance, enhancing thereby statistical power. We tested the group variable and the two group-wave interaction terms simultaneously on zero with a Wald test (Harrell, 2001), which we denote as global test. This global test combines information on the main effect of the group variable with differential trajectories over time into one statistic, which was used for primary screening on effects. In secondary analyses, we tested each component of the global statistics alone. Note that for the analyses of potential main effects of the group variable, the interaction effects were omitted. For sensitivity analyses, we adjusted all regression models additionally for medication (equivalent dosages (metric) and wave-dosage interaction term). To account for multiple testing, the Benjamini-Hochberg (BH) procedure was applied correcting for 272 regressions. A FDR < 0.05 was considered as significant. Post hoc, we explored the multivariate structure of the metabolome-wide significant metabolites showing the same longitudinal pattern in PD via a principle component analyses and examined the loading pattern on the first two principle components. For full results see Data S1.

To test the influence of PD on the statistical relation between two metabolites, mixed effect linear regressions were fitted, additionally including serially each metabolite and a metabolite-group interaction term into the model. We adjusted for age, sex, wave, group, and medication (equivalent dosages (metric) and wave-dosage interaction term). Here, the metabolite-group interaction term was the predictor of interest, capturing information on the question whether PD influences the statistical relation between two metabolites. This setup resulted in 272 times 271 regressions testing each pair of metabolites. Note that in comparison to analyzing ratios of concentrations, the applied procedure delivers exact p values for a change in statistical metabolite-metabolite relations in dependency on PD while avoiding the statistical problems accompanying the use of ratios (Hertel et al., 2018, Kronmal, 1993). BH correction was applied accounting for 73,712 tests.

#### Effects of medication

To explore the effects of PD medication on the metabolome, two analysis paradigms were applied. The first analysis paradigm investigated whether changes in dopaminergic medication dosages were associated with changes in individual metabolite concentrations in the PD group. To this end, we calculated the changes in metabolite concentrations between baseline and follow-up I, and between follow-up I and II, as well as the changes in equivalent dosages of dopaminergic medication. Then, we fitted mixed effect linear regressions with the change in metabolite concentration being the response variable and the change in equivalent dosages being the predictor of interest, including age, sex, length of disease, and the interval (baseline to follow-up I, follow-up I to follow-up II) as covariates. For controlling the FDR, BH correction was applied accounting for 272 tests. Post hoc, we examined the type of medication that was responsible for the metabolome-wide significant associations by testing the levodopa dosage, the total equivalent levodopa dosage, and the equivalent dosage of dopaminergic medication other than levodopa in subsequent analogous regressions.

Next, we performed association analyses using mixed effect linear on metabolite concentration levels, testing globally on associations with dopaminergic medication. Here, in a first set of regressions, levodopa equivalent dosages (levodopa dosage and equivalent dosage other than levodopa) were included as predictors of interest, while adjusting for age, sex, length of the disease, and wave. Second, the three most frequent drugs (levodopa, pramipexole, and rasagiline) were included as predictor of interests as binary variables (intake: yes/no) adjusting for the same covariates as before. All terms were tested simultaneously on zero via Wald tests to establish whether any linear combination of these three drugs, respectively dosages, could explain variance in metabolite concentrations. BH correction was applied accounting for 524 tests.

Third, we tested each drug in a separate set of regressions on association with metabolites in analogous regression models, correcting strictly for multiple testing. In terms of dosages, we tested for associations with levodopa dosage, total levodopa equivalent dosage, and levodopa equivalent dosages of dopamine receptor agonists. The equivalent dosage of MAO-B inhibitors was not included into this analysis, because of lack in variation. In terms of intake, we tested levodopa, pramipexole, and rasagiline. Thus, we corrected for 6^∗^272 = 1632 regressions using BH. The complete results are in the Data S1.

#### Associations with UPDRS scales

To analyze metabolite concentrations in association with the UPDRS in PD cases, mixed effect generalized linear models were created using the UPDRS scales as response variable and the log metabolite concentration as the predictor of interest. For the scales I (non-motor experiences of daily living), II (motor experiences of daily living), and IV (motor complications), we chose ordered logistic regressions to deal with the non-Gaussian distributions of the corresponding scores, while for the subscales III and the total sum of all scores linear regressions were applied. The models were adjusted for age, sex, wave, length of disease, and medication (equivalent dosages (metric) and wave-dosage interaction term). In primary analyses, we screened the total sum score for metabolomic associations, whereas in secondary analyses the subscales of the UPDRS were explored. Correction for multiple testing was applied for the primary analyses using BH accounting for 272 tests. The results of the secondary analyses were therefore only interpreted if a metabolite was significant regarding the total sum of the scales after correction for multiple testing. For full results, see Data S1.

#### Predictive metabolites for UPDRS scales at follow-up II

To test the metabolites associated with UPDRS scores on their potential predictive value, we performed generalized linear regression analyses as described above. We used the UPDRS scores at follow-up II as response variable and the log metabolite concentration at baseline as predictor of interest, while adjusting for age, sex, disease duration, medication status (levodopa intake and equivalent dosages) at follow-up I and baseline UPDRS scores. We focused on the subscale III (clinical motor examination), as the analyses above revealed that this scale was causative for the associations. These analyses were repeated for predicting follow-up II values with follow-up I characteristics and for predicting follow-up I values with baseline characteristics. The analyses were only performed for metabolites being significant after correction for multiple testing in the association analyses to the UPDRS scores above. As five metabolites were included into these analyses, we accounted for 15 tests in correction for multiple testing. In a second exploratory step, all subscales were analyzed as well as the sum of all subscales, resulting in 60 tests to be corrected for via BH procedure. The full results can be found in Data S1

#### Statistical analyses of individualized gut microbiota models and simulation results

We analyzed three statistical attributes of the personalized microbial community models, guided by the results of the metabolome analyses. First, microbiota abundances were compared on the species level by using fractional regressions (Papke and Wooldridge, 1996) with bootstrap derived standard errors based on 2000 replications. Fractional regressions are semiparametric generalized models designed for the analyses of relative frequencies without distributional assumptions. They can be parametrized in odds ratio, facilitating convenient interpretations of the regression coefficients. In these fractional regressions, the species abundance was the response variable and the group variable (PD versus control) the sole predictor. These analyses were primarily conducted for validating the reference mapping procedure. The results of significantly changing microbes between the groups were comparable to the original publication (Bedarf et al., 2017) and can be found in Data S1.

Second, for each personalized microbial community model, we analyzed the reaction abundances of all reactions involving homoserine and then all reactions in the pathway generating methionine from aspartate plus the reactions of the microbial transsulfuration pathway. In total, 28 reactions were tested, from which several reactions clustered together resulting in 22 independent statistical tests. Note that the reaction abundances were scaled by the relative abundances of the individual strains in the microbial community models. These pathways were targeted because of the metabolomic results identifying alterations in homoserine levels and metabolites along the transsulfuration pathway. The statistical analyses of reaction abundances were performed by using fractional regressions with the reaction abundance as response variable and the group variable (PD versus control) as sole predictor. To correct for multiple testing, BH correction was applied adjusting for 22 tests.

Next, we analyzed the secretion potentials of metabolites belonging to the described pathways via linear regressions with bootstrap-derived confidence intervals based on 2000 replications to get p values independent of distributional assumptions. The log secretion potentials were the response variables and the group variable (PD versus control) was the sole predictor. We tested eight secretion products, which exhibited variance in their secretion potentials and corrected for multiple testing accordingly using the FDR.

Finally, we calculated the contribution of the species *A. muciniphila* and *B. wadsworthia* in term of explained variance to the significant secretion potentials. This analysis was done using linear regressions with the log secretion potential as response variable and the log species abundance as predictor. As graphical inspection indicated non-linearity (Figure S3), we used fractional polynomials for modeling. Complete results on these analyses can be found in the ‘Data S1’ as well as Figure S3.

##### Statistical analyses of the EPIC-Norfolk data

We focused on taurine-conjugated bile acids in the statistical analyses of the EPIC-Norfolk cohort. Taurodeoxycholate, taurochenodeoxycholate, taurocholenate sulfate, taurocholate, tauro-beta-muricholate, taurolithocholate 3-sulfate, and tauroursodeoxycholate were covered. Each metabolite was tested separately using a Cox-model with the incidence of PD as outcome resulting in seven tests. Once again, we corrected for multiple testing using the FDR. All models were adjusted for age, sex, BMI, smoking status, and plasma concentrations of C-reactive protein as an inflammatory marker. Since metabolomics profiling was done in two large quasi-random batches, all analyses were performed for each batch separately and afterward meta-analyzed using fixed effect meta-analyses. Full results on these analyses including a measure of heterogeneity of results between the two batches can be found in the supplementary material (Data S1). Statistical analyses were performed in R (https://www.r-project.org/).

### Data and Code Availability

The metagenomics data from Bedarf et al. is deposited at the European Bioinformatics Institute-Sequence Read Archive database: ERP019674).

Application for access to EPIC Norfolk data or samples should be made via the study principal investigator Nick Wareham nick.wareham@mrc-epid.cam.ac.uk and the study co-ordinator nicola.kimber@mrc-epid.cam.ac.uk. Approved data requests will require a data sharing agreement between the applying research institution and the University of Cambridge. Links to contact information and request forms can be found here: http://www.mrc-epid.cam.ac.uk/research/studies/epic-norfolk/. More details on the Data Access & Sharing Policy can be found here: https://epi-meta.mrc-epid.cam.ac.uk/data_sharing_policy.shtml.

The datasets generated from the DeNoPa cohort supporting the current study have not been deposited in a public repository due to data protection laws for clinical data, but are available upon request submission, (brit.mollenhauer@paracelsus-kliniken.de), review, and approval of a research proposal outline. Note that, as patient data is involved, German and EU laws for data protection restrict public availability and have to be respected. Summary statistics of all analyses performed can be found in the supplemental data files.

The code for all statistical scripts and all the scripts for processing the metagenomics data is deposited at github under https://github.com/ThieleLab/CodeBase/tree/master/Scripts_Hertel_CellReports_2019. Genome-scale reconstructions for 773 microbes are available at the VMH database (www.vmh.life, v1.02). The COBRA Toolbox can be downloaded from https://opencobra.github.io/, and the Microbiome Modeling Toolbox (Baldini et al., 2019) is available at: https://github.com/opencobra/cobratoolbox/tree/master/src/analysis/multiSpecies/microbiomeModelingToolbox/.

## Supplemental Information

Document S1. Tables S1–S3 and Figures S1–S3

Data S1. Summary Statistics of All Performed Statistical Analyses, Related to Figures 1, 2, 3, and 4 Descriptive study statistics and extended results on metabolome analyses and microbiome modeling.

Document S2. Article plus Supplemental Information
