# Supplementary material for: Integrated Analyses of Microbiome and Longitudinal Metabolome Data Reveal Microbial-Host Interactions on Sulfur Metabolism in Parkinson’s Disease
Source: Cell Rep. 2019 Nov 12;29(7):1767–1777.e8. doi: 10.1016/j.celrep.2019.10.035 (PMC6856723; doi:10.1016/j.celrep.2019.10.035)
Supplement: Document S1. Tables S1–S3 and Figures S1–S3 [file mmc1.pdf]

Cell Reports, Volume *29*

Supplemental Information

Integrated Analyses of Microbiome and Longitudinal Metabolome Data Reveal Microbial-Host Interactions on Sulfur Metabolism in Parkinson's Disease

Johannes Hertel, Amy C. Harms, Almut Heinken, Federico Baldini, Cyrille C. Thinnes, Enrico Glaab, Daniel A. Vasco, Maik Pietzner, Isobel D. Stewart, Nicholas J. Wareham, Claudia Langenberg, Claudia Trenkwalder, Rejko Krüger, Thomas Hankemeier, Ronan M.T. Fleming, Brit Mollenhauer, and Ines Thiele

Table S1: Sample description DeNoPa cohort, Related to Star Methods (EXPERIMENTAL MODEL AND SUBJECT DETAILS)

	Controls	Cases	p-value
Age[a], mean (sd)	64.37(7.59)	64.00(8.49)	0.86[b]
Female, %	50%	50%	1.00[c]
Length of disease in months[a], median (interquartile-range)	-	12(9-24)	-
BMI, mean(sd)			
Baseline	27.59(4.68)	27.47(5.22)	0.926 [b]
Follow-up 1	27.54(4.96)	28.37(5.07)	0.522 [b]
Follow-up II	28.06(6.10)	27.83(4.69)	0.875 [b]
Serum creatinine, mean(sd)			
Baseline	0.84(0.029)	0.91(0.036	0.136 [b]
Follow-up 1	0.86(0.031)	0.92(0.038)	0.241 [b]
Follow-up II	0.86(0.031)	0.89(0.035)	0.473 [b]
Serum GGT, mean(sd)			
Baseline	35.24(5.01)	39.7(7.67)	0.629 [b]
Follow-up 1	35.7(4.81)	44.97(6.39)	0.251 [b]
Follow-up II	36.37(7.39)	50.21(10.97)	0.300 [b]
Total triglycerides, mean(sd)			
Baseline	120.10(9.29)	117.57(8.86)	0.844 [b]
Follow-up 1	143.91(14.31)	138.90(17.72)	0.827 [b]
Follow-up II	134.17(16.68)	117.89(11.66)	0.427 [b]
Levodopa, %			
Baseline	0%	10%	0.237[c]
Follow-up 1	0%	53.3%	<0.001[c]
Follow-up II	0%	83.3%	<0.001[c]
3-O-methyldopa levels[d], mean(sd)			
Baseline	0.18 (0.03)	1.20(0.8)	0.267 [b]
Follow-up 1	0.18 (0.03)	18.79(26.84)	<0.001 [b]
Follow-up II	0.19 (0.04)	30.34(28.67)	<0.001 [b]
UPDRS score, mean(sd)			
Baseline	1.2(1.73)	29.93(16.55)	<0.001 [b]
Follow-up 1	3.47(5.51)	35.83(21.42)	<0.001 [b]

Follow-up II	3.63(3.85)	40.73(21.07)	<0.001 [b]

BMI=body mass index, UPDRS=Unified Parkinson Disease Rating Scale, SD=standard deviation, GGT=Gamma-glutamyl-transferase

[a] at baseline

[b] p-value from welch t-test (robust against variance inhomogeneity)

[c] p-value from Fisher's exact test

[d] relative values against internal standard (unit-free)

Table S2: Longitudinal description of the UPDRS subscales. Related to Star Methods (EXPERIMENTAL MODEL AND SUBJECT DETAILS)

		Control group					Parkinson's disease group				
**Scale**	**Description**	**Baseline, mean(sd)**	**Follow-up I, mean(sd)**	**Follow-up II, mean(sd)**	**ICC**	**p-val***	**Baseline, mean(sd)**	**Follow-up I, mean(sd)**	**Follow-up II, mean(sd)**	**ICC**	**p-val ***
UPDRS_I	nonmotor experiences of daily living	.4(.968)	.867(1.961)	.867(1.252)	0,74	0,02245441	2.233(1.591)	2.733(2.651)	2.867(2.813)	0,42	0,76361817
UPDRS_II	motor experiences of daily living	.167(.379)	1.233(2.269)	1.033(1.299)	0,11	0,00495283	7.6(4.256)	9.567(5.532)	11.3(7.023)	0,4	0,02516821
UPDRS_III	motor examination	.633(1.066)	1(1.722)	1.1(2.04)	0,31	0,38386512	20(11.99)	21.83(14.02)	23.6(12.90)	0,58	0,25066236
UPDRS_IV	motor complications	0(0)	.367(.556)	.633(.615)	0,05	0,19991027	.1(.305)	1.7(1.685)	2.967(1.974)	0,27	2,1072E-07
UPDRS sum	total sum of the subscales	1.2(1.73)	3.467(5.507)	3.633(3.855)	0,39	0,00347403	29.93(16.55)	35.83(21.42)	40.73(21.07)	0,55	0,00688026

* p-value for change of scores over time from generalized linear (ordered logistic for scales I, II, and IV; linear for III and total sum) mixed effect regression models on sum scores with random intercepts for individuals
UPDRS=Unified Parkinson's Disease Rating Scale, SD=standard deviation, ICC= Intra-class correlation (estimates from mixed effect linear regression models)

Table S3: Extended results on associations of taurine conjugated bile acids and histidine with UPDRS scores, Related to Figure 3

Scale	Metabolite	OR per SD(95%-CI)	p-val main-effect	p-val wave-metabolite interaction terms	p-val (global test)
Nonmotor experiences of daily living	Glycochenodeoxycholate	1.01(.563,1.83)	0,958354592	0,720970213	0,867364049
	Taurodeoxycholate	1.03(.541,1.96)	0,927205324	0,580307126	0,77228117
	Taurolithocholate	1.13(.634,2.03)	0,667679131	0,418939978	0,577265501
	Taurochenodeoxycholate	.883(.477,1.63)	0,692703664	0,05119082	0,097403519
	Histidine	1.33(.772,2.30)	0,301499635	0,050487068	0,062795445
Motor experiences of daily living	Glycochenodeoxycholate	1.57(.888,2.79)	0,11999502	0,584910095	0,32993114
	Taurodeoxycholate	1.66(.885,3.12)	0,113711871	0,191467807	0,128062218
	Taurolithocholate	1.82(1.06,3.13)	0,029512389	0,770044744	0,162961528
	Taurochenodeoxycholate	1.09(.614,1.95)	0,756966412	0,000928063	0,002848896
	Histidine	.877(.514,1.49)	0,631691098	0,001826344	0,004779307
Motor complications	Taurodeoxycholate	1.16(.503,2.69)	0,721189737	0,507050216	0,681357145
	Taurolithocholate	1.43(.573,3.59)	0,440891027	0,718772769	0,71991986
	Taurochenodeoxycholate	.965(.387,2.40)	0,939195514	0,500429034	0,705573976
	Histidine	.797(.354,1.79)	0,583696365	0,534434259	0,694207132
		b per SD(95%-CI)	p-val main-effect	p-val wave-metabolite interaction terms	p-val (global test)
Motor examination	Glycochenodeoxycholate	**4.5414(2.1831,6.8998)**	**0,000160446**	0,752375066	**3,85941E-05**
	Taurodeoxycholate	**5.0123(2.4393,7.5853)**	**0,000134488**	0,459485054	**9,54299E-06**
	Taurolithocholate	**4.8466(2.5879,7.1054)**	**2,60499E-05**	0,455995142	**4,40051E-06**
	Taurochenodeoxycholate	**2.9977(.39110,5.6044)**	**0,024193915**	**0,020852594**	**0,000546943**
	Histidine	.10647(-2.902,3.1151)	0,944701612	**2,12226E-05**	**8,17327E-05**
Total sum	Glycochenodeoxycholate	**5.8812(2.4093,9.3530)**	**0,000899738**	0,888717771	**0,000527866**
	Taurodeoxycholate	**6.5909(2.5434,10.638)**	**0,001414916**	0,432464212	**0,000253226**
	Taurolithocholate	**6.3847(3.0044,9.7650)**	**0,000213873**	0,783009708	**0,000685799**
	Taurochenodeoxycholate	3.5190(-.3835,7.4216)	0,077174589	**0,003579476**	**0,000878645**
	Histidine	.21928(-4.262,4.7014)	0,923608303	**1,13654E-05**	**3,50652E-05**

SD=standard deviation, CI=confidence interval, UPDRS=Unified Parkinson's Disease Rating Scale.Estimates from mixed effect generalized linear regression (ordered logistic for subscales I,II and IV; linear for subscale III and total sum) with random intercepts for subject. All analyses adjusted for age, sex, length of disease, levodopa intake, levodopa dose and wave (categorical). In bold, FDR<0.05 for association with total UPDRS score, global test

OR=odds ratio, CI=confidence interval, SD=standard deviation, BL=baseline, FU1=follow-up I, FU2=follow-up II, UPDRS=Unified Parkinson Disease Rating Scale, TLCA=taurolithocholic acid, TCDCA=taurochenodeoxycholic acid, TDCA=taurodeoxycholic acid.

A PD-specific trajectories over time

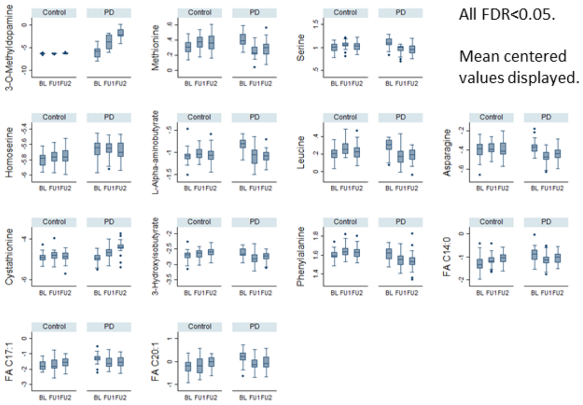

B Principle component analyses of the compounds showing decreasing concentrations over time in PD and no specific pattern in controls

Metabolite	PC1	PC2	Variance explained
Methionine	0.42	-0.13	71%
Serine	0.23	0.01	20%
Phenylalanine	0.34	-0.09	46%
Asparagine	0.34	-0.20	54%
Leucine	0.40	-0.09	65%
Alpha-aminobutyrate	0.40	0.21	73%
3-hydroxyisobutyrate	0.25	0.23	37%
FA C14:0	-0.03	0.45	54%
FA C17:1	-0.05	0.53	74%
FA C20:1	-0.00	0.54	76%

Variance explained denotes the variance explained for each metabolite concentration by the first two PCs.

C Differences between cases and controls per wave for metabolites with significant trajectories

Metabolite	Baseline		Follow-up I		Follow-up II	
	b(95%-CI)	p-val	b(95%-CI)	p-val	b(95%-CI)	p-val
3-methoxytyrosine	.38824(-.0348,.81130)	0,072078	2.6071(1.7212,3.4929)	8,01E-09	4.2203(3.5751,4.8655)	1,25E-37
Cystathionine	-.0127(-.2286,.20316)	0,907807	.13909(-.1030,.38120)	0,260166	.44752(.22520,.66984)	7,97E-05
Alpha-aminobutyrate	.25953(.11071,.40834)	0,00063	-.0405(-.1965,.11550)	0,610842	-.0221(-.1793,.13496)	0,782134
Asparagine	.04431(-.0354,.12412)	0,276413	-.0938(-.1723,-.0153)	0,019069	-.0429(-.1309,.04505)	0,338731
Homoserine	.16328(.08855,.23800)	1,85E-05	.10703(.03124,.18281)	0,005638	.05990(-.0221,.14193)	0,152393
Leucine	.09113(.01454,.16771)	0,019687	-.0827(-.1587,-.0067)	0,03285	-.0448(-.1172,.02749)	0,224185
Methionine	.10904(.02993,.18816)	0,006903	-.1092(-.1923,-.0260)	0,01006	-.0892(-.1616,-.0169)	0,015588
Phenylalanine	.10477(.04224,.16731)	0,001023	-.0143(-.0727,.04408)	0,630151	-.0337(-.0979,.03042)	0,302635
Serine	.10627(.01741,.19513)	0,019078	-.0977(-.1990,.00347)	0,058389	-.0568(-.1618,.04809)	0,288285
3-hydroxyisobutyrate	.08941(-.0511,.23002)	0,212647	-.1814(-.3602,-.0025)	0,046779	-.1702(-.3158,-.0247)	0,021844
FA C20:1	.43834(.21031,.66637)	0,000165	.08679(-.1547,.32833)	0,481278	.01349(-.2361,.26317)	0,915641
FA C14:0	.40975(.21019,.60931)	5,71E-05	.06578(-.1564,.28804)	0,561802	-.0400(-.2634,.18341)	0,725469
FA C17:1	.50203(.22941,.77465)	0,000307	.09557(-.1755,.36668)	0,48957	.06248(-.2205,.34553)	0,665258

Estimates from mixed effect linear regression with random intercepts for subject. All analyses adjusted for age, sex. Group-wave interaction terms parametrized as one categorical variable having six categories.

D Trajectories for the two PCs representing metabolites with decreasing values in PD

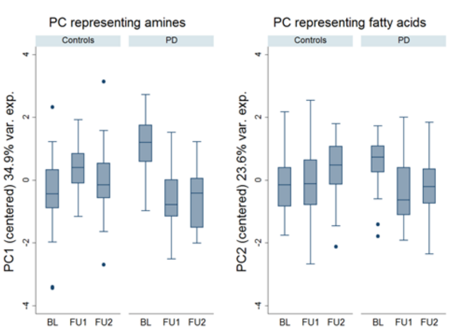

Both trajectories were PD-specific (PC1: p=3.482e-08, PC2: p=0.0001).

Figure S1: PD-specific trajectories over time, Related to Figure 2. A Box plots of log transformed metabolite concentrations over the three waves for controls and PD cases. Metabolite concentrations are displayed after mean centering per individual, showing therefore the intra-individual variation over the waves for controls and PD cases. **B** Principle component analyses of the compounds showing decreasing concentrations over time in PD and no specific pattern in controls. **C** Differences between cases and controls per wave for metabolites with significant trajectories. **D** Trajectories for the two PCs representing metabolites with decreasing values in PD. Both trajectories were PD-specific (PC1: p=3.482e-08, PC2: p=0.0001). FA=fatty acids, BL=baseline, FU1=follow-up 1, FU2=follow-up 2, PC=principle component.

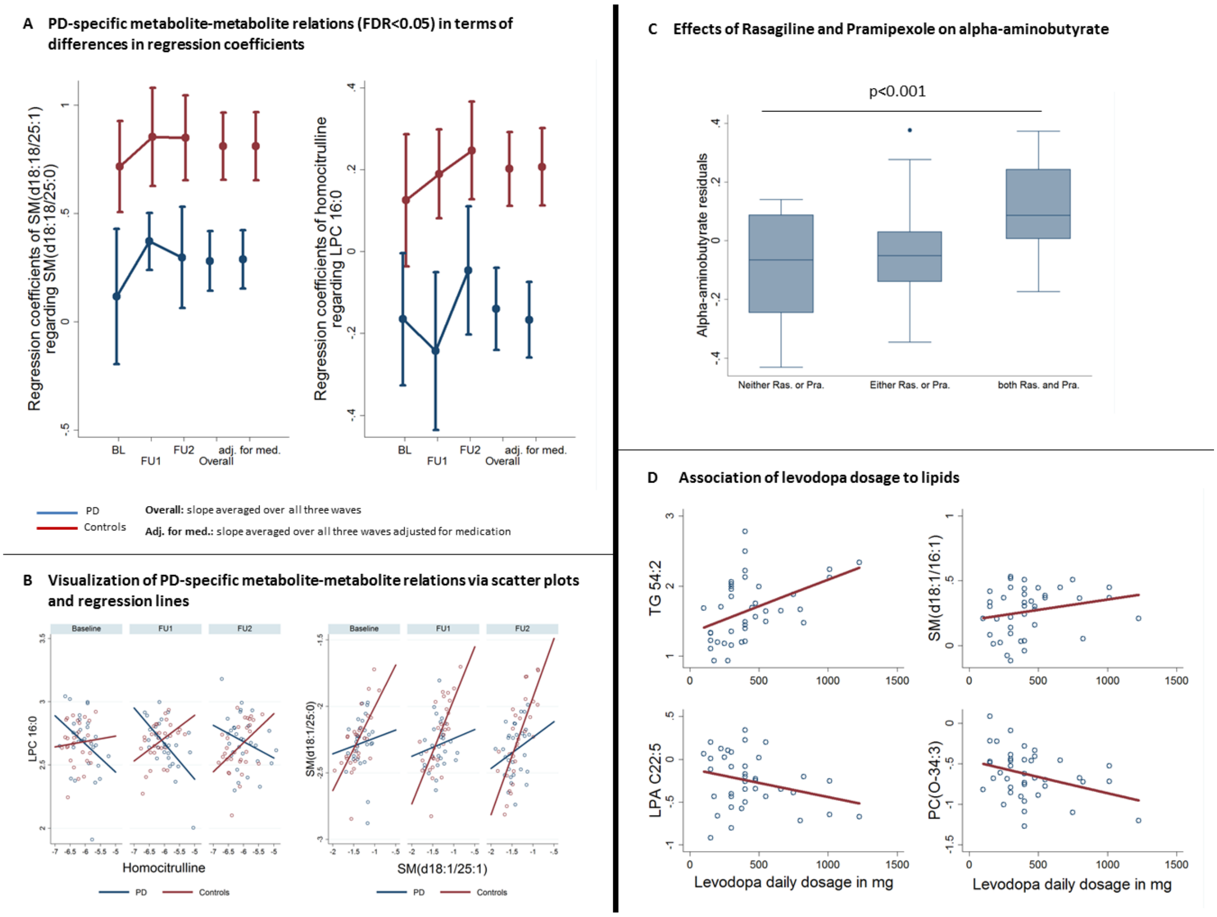

Figure S2: Extended results regarding metabolite-metabolite relations and effects of dopaminergic medication, Related to Figure 3. **A** Altered metabolite-metabolite associations with FDR<0.05. The regression coefficients (adjusted for age, sex, wave, group and wave-group interaction terms) and their confidence intervals are depicted for each wave, and averaged over all three waves. Adjustment for medication includes the levodopa equivalent dosage, pramipexole, rasagiline and levodopa intake. **B** Scatter plots for LPC 16:0 in dependency on homocitrulline, and SM(d18:1/25:0) in dependency on SM(d18:1/25:1) with corresponding regression lines stratified for wave. Red indicates control, blue PD. **C** Combined effects of Rasagiline and Pramipexole on alpha-aminobutyrate levels. **D** Association of levodopa dosages to diverse lipids displayed by scatter plots and corresponding regression lines. All associations were significant after multiple testing FDR<0.05. SM=Sphingomyelin, LPC=lysophosphatidylcholine, TG=triglycerides, LPA=lysphosphatic acid, PC=phosphatidylcholine, BL=baseline, FU1=follow-up 1, FU2=follow-up 2.

A Tested secretion potential of microbial communities regarding differences between PD and controls

Secreted metabolite	b(95%-CI)	p-value	FDR
Methionine	.49845(.25013,.74676)	8,34E-05	0,000667
Hydrogen sulfide	.57589(.20729,.94449)	0,002197	0,008789
Sulfite	.81821(.22327,1.4131)	0,007028	0,018742
Asparagine	-.0704(-.1277,-.0131)	0,016029	0,032059
Aspartate	.08454(-.0265,.19561)	0,135708	0,217133
Serine	-.0420(-.1039,.01988)	0,183381	0,244508
Cysteine	.00901(-.1756,.19367)	0,923814	1
Glutathionine	.00003(-.0005,.00059)	0,916499	1

CI=confidence interval, FDR=false discovery rate
Estimates from linear regressions using the group variable as the sole predictor with bootstrap-derived confidence intervals using 2000 replications.

B Log *Akkermansia muciniphila* abundance and log secretion potentials of methionine, hydrogen sulfide, asparagine and sulfate

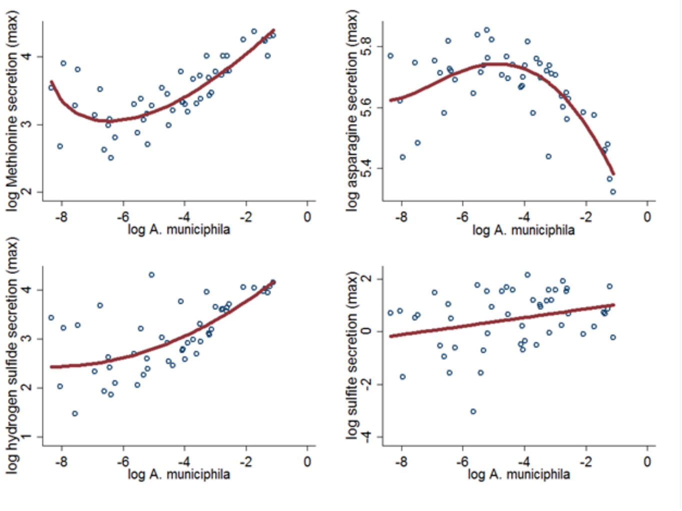

Figure S3: Extended results of metagenomics analyses, Related to Figure 4. A Secretion potentials in transsulfuration pathway from metabolic modeling of metagenomic data. **B** Secretion potentials in dependency on *akkermancia municiphila* abundance. Red line shows the polynomial fit.
